# Body Weight Misperception and Dissatisfaction Among Overweight and Obese Adult Nigerians

**DOI:** 10.2196/publichealth.7047

**Published:** 2017-08-16

**Authors:** Mukadas Oyeniran Akindele, Joliana Phillips, Ehimario Igumbor, Ushotanefe Useh

**Affiliations:** ^1^ Diseases of Lifestyle Niche Area School of Research and Postgraduate Studies North-West University Mafikeng South Africa; ^2^ Department of Physiotherapy Faculty of Allied Health Sciences Bayero University Kano Nigeria; ^3^ Department of Physiotherapy University of the Western Cape Cape Town South Africa; ^4^ School of Public Health Faculty of Community and Health Sciences University of the Western Cape Cape Town South Africa

**Keywords:** overweight, obesity, misperception, Nigeria, adult

## Abstract

**Background:**

The increase in the prevalence of overweight and obesity in low- and medium-income countries has a negative impact on overall health. Correct perception of one’s body weight is a step in seeking healthy help toward weight reduction in overweight and obese individuals.

**Objective:**

This study was carried out to assess the body weight misperception and dissatisfaction among overweight and obese adults in an urban African setting.

**Methods:**

This study was part of a larger cross-sectional study that was designed to plan an intervention for overweight and obese adults in an urban African setting. For this study, we randomly selected only overweight and obese adults (≥18 years old) who consented to participate in the study from 15 enumeration areas in Alimosho Local Government Area of Lagos State, Nigeria. We followed the World Health Organization guidelines for conducting community surveys in recruiting overweight and obese participants. We assessed body weight perception and dissatisfaction through their responses to the following: “How do you describe your weight?” and “I feel bad about myself because of my weight.” Data for this study were collected between November 2012 and March 2013.

**Results:**

We recruited 567 participants, of whom more than half (n=304, 53.6%) misperceived their weight as either underweight or normal weight, and 61.2% (n=186) of whom were women. The strength of agreement between the actual body mass index and weight perception was very poor (κ=.032, SE .015, *P*=.04). The strongest predictor of weight perception was sex (female) with an odds ratio of 1.63 (95% CI 1.13-2.35). About 41.1% (n=233) of the participants were dissatisfied with their weight, of whom 30.0% (n=70) were men. Age (young adult) was a predictor of weight dissatisfaction with an odds ratio of 2.37 (95% CI 1.62-3.46).

**Conclusions:**

More than half of the participants misperceived their body weight as either underweight or normal weight, and the majority of them were women. More men were not happy with their body weight, and participants in the young adult age group were more dissatisfied with their body weight.

## Introduction

Weight perception is a concept of how an individual perceives his or her weight appropriateness. Self-perceived weight has been documented to have positive associations with effective weight control and weight loss behaviors in adults [1]. Appropriate weight perception is of utmost importance, as this would stimulate the need to reduce weight by individuals who are either overweight or obese [2]. Some overweight and obese individuals do misperceive their body weight. This misperception of weight can hinder the prevention, control, and management of overweight and obesity [2,3]. Weight misperception is the disagreement between an individual’s actual weight status and the person’s perception of his or her weight [4], which can be categorized into body weight underestimation and body weight overestimation. Weight underestimation is a situation in which overweight individuals consider themselves to be underweight whereas they are overweight, while weight overestimation is when individuals with normal or underweight considers themselves to be overweight as determined by body mass index (BMI) [5].

Weight misperception has been a public health concern, since it can result in large numbers of overweight and obese individuals failing to understand the need for weight control or losing weight [6]. This will eventually affect interventions toward overweight prevention, control, and management. It has been hypothesized that weight misperception among overweight and obese individuals might deter their adoption of healthy weight reduction behaviors [7]. Subjective evaluation of health has been shown to have a link with misperception [8]. The discordance between actual body weight and perceived body weight is associated with depression [8,9], inappropriate weight control practices [8,9], and negative body image [9], which are precursors of health-related quality of life impairments [10]. Weight misperception has been reported in the literature among the youths and adults of different countries. A high prevalence of weight misperception has been reported among youth in Pakistan, a developing country, with 42.4% overall weight misperception seen in the total youth population [11]. High prevalences of weight misperception have also been reported in Spain [12], the United States [5], and China [13].

Little is known about weight misperception among overweight and obese individuals in the urban setting of sub-Saharan Africa. Ethnic and racial differences in body weight perception have been reported in the literature. Non-Hispanic blacks and Mexican Americans who are overweight or obese have been found to view themselves as underweight and incorrectly perceived themselves to be at the recommended weight [14]. Duncan et al [4] also reported that nearly one-quarter of the US sample of overweight and obese adults misperceived their body weight. Also, it has been reported that body weight misperception among overweight individuals was more common among blacks than among whites and less common in women than in men [15]. Among rural dwellers in Nigeria, Akinpelu et al [16] reported that a large proportion of participants in their study could not perceive their weight accurately. Appreciating the issues of weight misperception might increase the awareness of the need to reduce body weight among overweight and obese persons [14], thereby enhancing healthy life behavior and successful weight reduction. We carried out this study to determine the prevalence of weight misperception among overweight and obese Nigerians.

## Methods

### Participants

This was part of a cross-sectional study carried out at Alimosho Local Government Area of Lagos State, Nigeria. Alimosho Local Government Area has a population of over 1,277,714 (2006 National Population Census) [17]. Using the World Health Organization (WHO) [18] guidelines for conducting community surveys, we randomly chose 5 of 11 political wards into which Alimosho area is divided. We randomly selected 3 census enumeration areas in each of the 5 chosen political wards in Alimosho Local Government Area through National Population Commission 2006 census enumeration areas. We selected houses with odd numbers for survey in each census enumeration area and recruited participants who were 18 years of age and older. The participants for this study were overweight or obese individuals who gave written informed consent to participate in the whole cross-sectional study.

We administered the WHO STEPS Questionnaire to each of the participants [19]. Not all of the data gathered from the STEPS Questionnaire were relevant for this study; we used a section of the STEPS Questionnaire pertaining to demographic information. The demographic data we collected were sex, age, years spent at school, highest educational level attained, racial group, marital status, work status, number of people in the household, and income. To measure height, we instructed each participant to stand barefoot with feet together on a level cemented floor, buttocks and heels touching the wall, head held erect, and eyes looking forward so that the lower margin of the external auditory canal was in the Frankfurt horizontal plane. The point of greatest height to the nearest 0.1 cm was marked off on the wall with a stretch‐resistant tape. For weight measurement, we encouraged each participant to put on minimal clothing material prior to measurement. The participant’s weight was measured using a Tanita BC-549 Plus Ironman body composition monitor (Tanita Europe BV, Amsterdam, the Netherlands). The weight was recorded to the nearest 0.5 kg. To ensure reliability of these measurements, we took 3 measurements of the participant’s height and weight and used the average of the 3 measurements.

### Body Weight Perception and Dissatisfaction Measurement

We assessed body weight perception and dissatisfaction mainly through 2 questions in the baseline survey using the protocol of Wang et al [20]. Responses to “How do you describe your body weight? were scored as follows: underweight=1; normal weight=2; a little overweight=3; very overweight=4. Responses to “I feel bad about myself because of my weight” were scored as follows: very true=1; little true=2; not true=3; can’t say=4.

We obtained permission and ethical clearance from the University of the Western Cape Research Ethics Committee (12/9/15) and Lagos State University Health Research and Ethics Committee (LREC/10/06/261).

### Statistical Analysis

We recorded frequencies and percentages for categorical variables. For ease of analysis, we recoded the sociodemographic variables age (young adults, middle-aged adults, and older adults), educational status (primary school completed, secondary education completed, tertiary education completed, and postgraduate education), employment (employed, unemployed, and pensioner), and marital status (married and single). We assessed the relationship between independent (sociodemographic) variables and weight perception and weight dissatisfaction using chi-square analysis, and subjected only the variables that showed a significant relationship to logistic regression. We assessed the predictors of the relationship between independent (sociodemographic) variables and weight perception and weight dissatisfaction using logistic regression. Kappa statistics were used to determine the strength of agreement between weight perception and actual body weight (BMI). We set the level of significance at .05. We analyzed the data using IBM SPSS version 23 (IBM Corporation).

## Results

We administered the body weight perception questionnaires to a total of 567 overweight and obese Nigerians. Of these, 193 (34.04%) were men and 374 (65.96%) were women. The strength of the agreement between BMI and weight perception was very poor among our participants (κ=.032, SE .015, *P*=.04).

### Weight Misperception by Overweight and Obese Nigerians

Table 1 shows the weight status of our sample. As Table 2 shows, of all respondents misperceiving their weight as normal, 38.6% (112/290) were men and 61.4% (178/290) were women. Also, among those who misperceived their weight as a little overweight, 72.7% (141/194) were women and 27.3% (53/194) were men. It should be noted that the participants in these studies were overweight and obese adults. Those participants who classified themselves as either underweight or normal weight actually misperceived their actual weight status. There was no significant difference between male and female weight perception (χ^2^_3,n=567_=7.24, *P*=.07).

### Predictors of Weight Perception by Overweight and Obese Nigerian Adults

We carried out a binomial logistic regression to determine which of the sociodemographic variables predicted weight perception. Our model contained sex, age, marital status, employment status, and educational status as predictors. We observed that the full model containing all sociodemographic predictors was statistically significant (χ^2^_9,n=567_=25.60, *P*<.001), showing that our model was able to distinguish between weight perception respondents. However, only 3 of these predictors (female, middle-aged adult, and unemployed) made a statistically significant input to our model, as Table 3 shows. The strongest predictor of weight perception was sex (female), with an odds ratio of 1.63, indicating that women were 1.63 times more likely to misperceive their weight. The odds ratio of 0.39 (which is less than 1) for unemployed participants shows that they were less likely by 0.39 times to misperceive their weight (Table 3).

**Table 1:** Body mass index (BMI) status of the study population.

BMI category	Men n (%)	Women n (%)	Total n (%)
Overweight	142 (73.6)	165 (44.1)	307 (54.1)
Obese	51 (26.4)	209 (55.9)	260 (45.9)
Total	193 (34.0)	374 (66.0)	567 (100)

**Table 2:** Weight misperception by overweight and obese Nigerians.

Response	Men n (%)	Women n (%)	Total n (%)
Underweight	6 (42.9)	8 (57.1)	14 (100)
Normal weight	112 (38.6)	178 (61.4)	290 (100)
A little overweight	53 (27.3)	141 (72.7)	194 (100)
Very overweight	22 (31.9)	47 (68.1)	69 (100)
Total	193	374	567

**Table 3:** Relationship between body weight misperception and demographic variables.

Sociodemographic characteristics		B	SE	Wald	*df*	*P* value	Odds ratio	95% CI
**Sex**								
	Male (reference)							
	Female	0.490	0.186	6.962	1	.008^a^	1.633	1.134-2.350
**Age**								
	Young adult (reference)							
	Middle-aged adult	–0.667	0.191	12.219	1	.001^a^	0.513	0.353-0.746
	Older adult	–0.467	0.353	1.750	1	.19	0.627	0.314-1.252
**Educational status**								
	Primary school completed (reference)							
	Secondary school completed	–0.277	.366	0.573	1	.45	0.758	0.370-1.553
	Tertiary education completed	–0.125	.357	0.123	1	.73	0.882	0.439-1.776
	Postgraduate education	0.023	.369	0.004	1	.95	1.023	0.497-2.107
**Employment status**								
	Employed (reference)							
	Unemployed	–0.937	.446	4.409	1	.04^a^	0.392	0.164-0.940
	Pensioner	–0.877	.841	1.086	1	.30	0.416	0.080-2.164
**Marital status**								
	Single (reference)							
	Married	–.147	.254	0.335	1	.56	.863	0.525-1.420
Constant		0.124	0.397	0.097	1	0.76	1.132	

^a^Significant at *P*<.05.

### Weight Dissatisfaction Among Overweight and Obese Nigerian Adults

Table 4 shows how dissatisfied our participants were regarding their weight. Descriptive statistics show that 233 (41.1%) of all participants were not happy with their weight, of whom 70 (30.0%) were men and 163 (70.0%) were women. However, there was a significant difference between how men and women felt about their weigh (χ^2^_3,n=567_=16.53, *P*=.001).

### Predictors of Weight Dissatisfaction Among Overweight and Obese Nigerian Adults

We performed logistic regression to assess the impact of sociodemographic variables on weight dissatisfaction among the overweight and obese adult Nigerians in our study population. The model contained sex, age, highest educational level, employment status, and marital status as independent variables, with weight dissatisfaction as the dependent variable. The model explained between 4.7% (Cox and Snell *R*^2^) and 6.4% (Nagelkerke *R*^2^) of the variance in weight dissatisfaction and correctly classified 62.4% of cases. Logistic regression also showed that the independent variable age made a unique statistically significant contribution to the regression model (χ^2^_9,n=567_=27.40, *P*=.001), which implies that the model was able to distinguish between overweight and obese adults who were able to show dissatisfaction with their weight correctly and incorrectly. The main predictor of weight dissatisfaction was age (young adult) with an odds ratio of 2.37. This can be interpreted as overweight and obese young adults being 2.37 times more dissatisfied with their body weight.

**Table 4:** Weight dissatisfaction among the overweight and obese Nigerian adult study population.

Description	Men n (%)	Women n (%)	Total n (%)
Dissatisfied	70 (30.0)	163 (70.0)	233 (42.9)
Satisfied	123 (37.9)	211 (65.1)	324 (57.1)
Total	193 (34.0)	374 (66.0)	567 (100)

## Discussion

### Principal Findings

This study sought to determine the prevalence of weight misperception and dissatisfaction among Nigerian overweight and obese adults, as well as those factors that would predict weight perception and dissatisfaction. The outcome of this study shows that more than half of our participants perceived their BMI as either underweight or normal, and the majority of them were women, although we found no difference between male and female weight perception. The level of agreement between BMI and weight perception was actually very poor, which accounts for why more than half perceived their weight to be underweight or normal. Of the participants, 41.1% displayed dissatisfaction toward their body weight (BMI).

Weight reduction success depends on a few factors, among which are weight control practice and behavior acquired by the individual. Recognition and appreciation of one’s body weight once it is compromising health is an important factor in reducing excess weight. This can be achieved through accurate body weight perception. Weight control behaviors and practices have been shown to be caused by body weight perception [20].

Weight misperception was reported among overweight and obese Sri Lankan adults in a study in which more than two-thirds of overweight and one-third of obese Sri Lankan adults misperceived their weight to be normal or underweight [21]; the majority of them were women. This is in line with the findings of our study. Although this study was carried out among Nigerian adults, similar results were reported among adolescents in Hong Kong [22]. While looking at differences in perceived weight and attractiveness among overweight black or white America women, Chithambo and Huey [23] found that black women reported lower perceived weight than did white women. Our study showed very poor agreement between actual body weight (BMI) and perceived body weight. Since self-perception of body weight is a major determinant of nutritional practices and weight management [24], health care professionals and public health physicians should step up awareness campaigns regarding the importance of accurate body perception.

The predictors of weight misperception were sex (female), young adult age, and being employed. This is contrary to the finding of Jayawardena et al [21], who reported that older age was a significant predictor of underperception of body weight. This difference might arise from their use of self-reported weight and height to arrive at their participants’ BMI, which had been earlier reported to be unreliable and inconsistent [25]. Also, the weight perception questionnaire administered to our participants was different from that used by Jayawardena et al [21], whose body weight perception question contained 5 options, whereas ours contained 4 options. Furthermore, the participants in the Jayawardena et al [21] study had participated previously in the Sri Lankan Diabetes Cardiovascular Study (SLDCS), whereas the participants in our study had never participated in this type of study. Findings from Jayawardena et al [21] might have been influenced by recall or learning effects, since they had participated previously in a similar study [26]. However, the findings in our study were similar to those of Chang and Christakis [12] who reported that age, sex, and income were independently associated with self-evaluation of weight status in an American population. Furthermore, age and sex (female) were also reported to be associated with weight misperception among overweight and obese adults in Pakistan [27].

The extent of body weight satisfaction or dissatisfaction hinges on self-assessment of one’s body, the knowledge of which is personal and cannot be assessed or determined by someone else [28]. Body weight dissatisfaction might play a role in healthy weight behaviours and practices. Less than half of the participants in our study reported body weight dissatisfaction, and age is a predictor of body weight dissatisfaction. Similar results were reported among Israeli Arab women by Niskar et al [29] and among overweight and obese adult women in the United States [30]. Body weight dissatisfaction might be influenced by racial and cultural affiliations, especially among the black population in sub-Saharan Africa [31]. There is an unconfirmed belief that a bigger and plumper body size attracts respect and indicates the absence of diseases in some cultural settings. A cross-cultural perspective study to determine the weight control behaviour among women of diverse ethnic affiliations, however, reported that larger body size is more acceptable to women of ethnic minorities, and hence mitigates weight reduction behaviour [32]. In Nigeria, the social and cultural acceptability of overweight and obesity has been reported in the literature [33]. Marital preference is given to individuals who are bigger because it connotes affluence and healthiness. This might hinder healthy weight and weight reduction practices and behaviors. In view of these, the need arises to change cultural orientations and perceptions of plumpness as a sign of affluence to being a sign of ill health by public health and medical practitioners.

### Conclusion

This study has shown that the majority of participants misperceived their body weight (BMI) and less than half of the participants were dissatisfied with their body weight. These results should be interpreted and generalized with caution because we obtained the responses subjectively. Another limitation is that the 2 questions that assessed weight perception and dissatisfaction were not validated and their reliability was not determined. However, the strength of this study lies in the fact that it was conducted on only overweight and obese adults of both sexes. We recommend, therefore, that further studies should be carried out using a validated measure to assess weight misperception and dissatisfaction. We also recommend that various weight reduction steps and attempts being made by overweight and obese individuals should be assessed.

## Abbreviations

BMI: body mass index
SLDCS: Sri Lankan Diabetes Cardiovascular Study
WHO: World Health Organization
